# Spatial–temporal distribution of total organic carbon and its transportation in the Jiulong River Estuary

**DOI:** 10.1038/s41598-022-13268-0

**Published:** 2022-06-07

**Authors:** Cui Wang, Yi Ding, Zhouhua Guo, Hui Lin, Junwen Wu

**Affiliations:** 1 Third Institute of Oceanography, Ministry of Natural Resources, Xiamen, 361005 China; 2 Guangdong Provincial Key Laboratory of Marine Disaster Prediction and Prevention & Institute of Marine Sciences, Shantou University, Shantou, 515063 China; 3 Southern Marine Science and Engineering Guangdong Laboratory, Guangzhou, 511458 China

**Keywords:** Environmental sciences, Environmental impact

## Abstract

Spatial and temporal distributions of total organic carbon (TOC) in the Jiulong River Estuary (JRE) were determined using data collected during three cruises in summer 2010, autumn 2010, and spring 2011. The TOC concentration influencing factors were identified, and the export fluxes were calculated. TOC concentration ranges were 0.73–4.17 mg/L in summer, 0.90–5.32 mg/L in autumn, and 1.78–8.03 mg/L in spring, respectively. TOC concentrations of the surface water and nearshore area were higher than those of the bottom water and offshore area, respectively, and the maximum TOC content occurred in the JRE upper reaches. The TOC concentration decreased with increasing salinity and exhibited a significant positive correlation with petroleum and dissolved inorganic nitrogen (DIN), indicating the influence of terrestrial input. A weak relationship between TOC and chlorophyll-a indicated that phytoplankton was not the dominant source of TOC. TOC fluxes discharged into the JRE were 50.39 × 10^3^ t/a in 2010 and 46.08 × 10^3^ t/a in 2011, and those transported into the sea were 38.46 × 10^3^ t/a in 2010 and 33.66 × 10^3^ t/a in 2011, respectively, accounting for approximately 75% of the total estuary fluxes. This study elucidates the biogeochemical processes of estuarine organic carbon and provides a quantitative basis for the land–sea integration of carbon dioxide emission reduction and sink increase projects.

## Introduction

Since the industrial revolution, the concentration of greenhouse gas carbon dioxide (CO_2_) in the atmosphere has significantly increased, resulting in outstanding global warming. The Chinese government is implementing a double carbon emission reduction plan in response to global warming, with a carbon peak in 2030 and carbon neutralization in 2060. The primary pathways to achieve carbon neutrality include CO_2_ emission reduction and sink increase. Therefore, an in-depth study to understand the global marine carbon cycle is necessary. The ocean is the largest active carbon pool on the earth, and it exhibits an enormous potential for negative emissions^1^. Although estuarine and marginal seas account for only 7–10% of the global ocean area, they contribute to 30–50% of the global ocean primary production and 80% of the organic carbon burial^2,3^, and they play a vital role in the global carbon cycle^4^. Estuaries are critical channels that connect terrestrial and marine ecosystems. Compared with open ocean and marginal shelf seas, estuaries are highly affected by runoff and tidal currents and their biogeochemical processes are complex, rendering them one of the most complex areas for studying the carbon cycle^5^.

Estuaries are not only channels for the entry of terrigenous materials into the sea, but they are also facilitators. Biogeochemical processes in estuaries add or remove carbon, changing the final marine flux and material composition of the water. Of the estimated 1.0 Pg C a^−1^ of the carbon flux transported into rivers since the industrial revolution^6^, 40% (~ 0.4 Pg C a^−1^) has returned to the atmosphere, 50% (~ 0.5 Pg C a^−1^) has been buried in sediments, and 10% (~ 0.1 Pg C a^−1^) has entered the ocean. Such a large input of organic carbon indicates estuaries are hotspots of mixing, transporting, and transforming organic carbon, inevitably impacting the estuarine carbon cycle. Estuaries are affected by multiple factors, such as land use and human activities, resulting in significant spatial and temporal variabilities in the carbon flux into the sea^4,7^. Therefore, there is a need for an in-depth study of river-estuary–sea organic carbon transport characteristics, spatial–temporal variabilities, and influencing factors to clarify the biogeochemical processes of organic carbon in estuaries and the role of the estuarine carbon cycle. This information can provide a quantitative basis for land–sea integrated CO_2_ emission reduction and sink increase projects^8,9^.

The Jiulong River, located in the south of the Fujian Province, flows into the Jiulong River Estuary (JRE). It is the second–largest river in the province, with a basin area of approximately 14,741 km^2^, accounting for approximately 12% of the province land area. The Jiulong River Basin has a subtropical marine climate, warm and humid, and abundant rainfall, with annual average precipitation of 1684.4 mm^10^. The JRE is a typical shallow estuary connecting the Xiamen Bay and the Taiwan Strait. It covers an area of approximately 100 km^2^, and its depth is 2–10 m (average depth of 4 m)^11^. The JRE is approximately 21 km long from east to west and 6.5 km wide from north to south^12^. Owing to economic development, a substantial amount of nitrogen and phosphorus pollutants brought by the Jiulong River have entered the JRE, and the pollution load of the estuary area has been steadily increased. The nutrient fluxes have increased sharply from 3.8 × 10^3^ t/yr to 43.6 × 10^3^ t/yr for dissolved inorganic nitrogen (DIN) and from 0.091 × 10^3^ t/yr to 1.1 × 10^3^ t/yr for soluble reactive phosphorus from the 1980s to the 2010s^12^. The degree of eutrophication has intensified, and harmful algal bloom events have occurred frequently during 1990–2020^13^. Additionally, the Haicang and Zhanyin ports are located on the north and south sides of the JRE, resulting in a large volume of oil-bearing wastewater near the port areas. These oil–bearing wastewaters are a potential source of organic carbon. Nitrogen and phosphorous pollution and heavy metal sources in the JRE have been previously studied^14,15^. Liu et al.^16^ studied the variations in dissolved carbon in the Jiulong River to understand the effects of carbonate rock weathering, climate change, phytoplankton, and human activities on dissolved carbon concentrations in rivers. Qiao et al.^17^ traced changes in particulate organic carbon (POC) sources and fluxes in the Jiulong River during a rain event and suggested that hydrology played a critical role in exporting terrigenous POC.

Located in between the land and ocean, estuaries are complex dynamic systems subjected to significant seasonal changes^18^. However, owing to the lack of field data from representative estuaries worldwide, great uncertainties remain in the measurements of TOC and derived emissions from estuaries. To date, direct studies on the spatial and temporal distributions and transport of organic carbon in estuaries and inshore waters have focused on large estuaries in temperate regions, such as the Mississippi River Estuary^19^, Thames River and Rhine Estuary^20^, and Yellow River Estuary^21,22^, and tropical (subtropical) regions, such as the Amazon River Estuary^23^, Yangtze River Estuary^24,25^, and Pearl River Estuary^1,26^. The concentration of organic carbon varies greatly in estuaries globally owing to differences in geographical conditions and the influence of human activities^19,27^. However, few studies have been conducted on the distribution and transport of organic carbon in medium–sized rivers and estuaries in subtropical regions that are significantly affected by human activities, such as the JRE. In addition, it remains unclear how organic carbon is transported from the river to the sea by estuarine dynamics under estuarine hydrologic conditions. Based on three seasonal campaigns in 2010 and 2011, we analyzed the spatial–temporal distribution of TOC in the spring, summer, and autumn and evaluated its transport characteristics and influencing factors to provide a scientific basis for further understanding of TOC biogeochemical processes in the JRE.

## Materials and methods

### Sampling strategy

**Figure 1:**
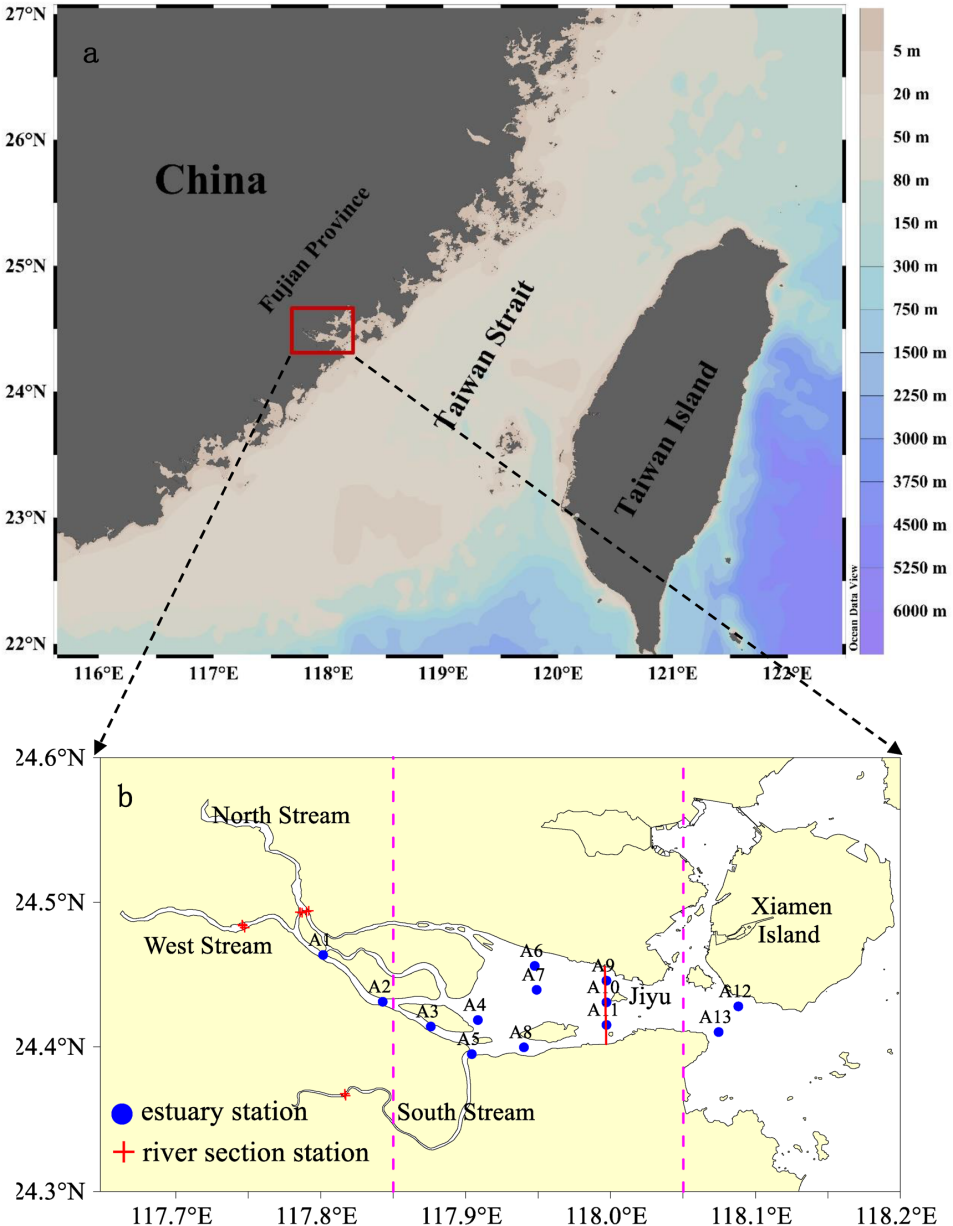
Maps of the (**a**) investigated area and (**b**) sampling stations in the Jiulong River Estuary (JRE). The satellite imagery in figure (**a**) was obtained from and drawn using the free software Ocean Data View (ODV 5.1.2) (Schlitzer, Reiner, Ocean Data View, https://odv.awi.de, 2018); figure (**b**) was created with Surfer, Version 10 (https://www.goldensoftware.com/products/surfer). Red dotted lines indicate the rough boundaries of the upper, middle, and lower reaches of the JRE.

Three survey cruises were conducted in the JRE in August 2010, November 2010, and May 2011. Thirteen sampling stations were conducted along the JRE salinity gradient for each cruise (Fig. 1). Each survey was conducted in the high and low tides during the spring tide period. Surface and bottom water samples were collected, according to the water depth. Samples were analyzed for temperature, salinity, TOC, DIN, chlorophyll-a, petroleum. To understand the water quality of the Jiulong River, sampling monitoring was collected at four stations in the North Stream, three stations in the West Stream, and two stations in the South Stream. In August 2010 and May 2011, diurnal tide observation stations (A9, A10, A11) were assembled near the Jiyu section (Fig. 1), and water samples were collected every 2 h over one complete tidal cycle to observe the tidal variations in TOC concentration. To study the TOC flux into the sea, a north–south section was set near the Jiyu section of the JRE in August 2010, and diurnal tidal observations using acoustic doppler current profiler (ADCP) navigation were conducted to obtain the section hourly flux.

### Sample handling and analysis methods

Following the Specification of Oceanographic Survey (GB 17378.4-2007)^28^, the TOC concentration was determined using the high-temperature combustion catalytic oxidation method and a Shimadzu TOC-V_CPH_ analyzer. The acidified water sample was added into the injector, the device was then rinsed with the water sample three times (approximately 3 mL each time), and finally, 1 mL was left for determination. After the water sample was fully aerated with high-purity oxygen for 2–3 min (to remove the dissolved inorganic carbon), it entered a 680 °C high-temperature combustion tube equipped with a catalyst. The organic carbon was then combusted and converted into CO_2_. Then, the CO_2_ was transferred with the carrier gas through the condensation well, and then it entered a non-dispersive infrared detector for detection. Each sample was assessed 3–5 times. The instrument setting condition was a standard deviation of several determination results of the same sample of less than 0.1 or a coefficient of variation of less than 2%.

To determine the DIN concentration, approximately 1 L of seawater samples was filtered using a 0.45 µm polycarbonate filter membrane and then analyzed within 6 h. The concentration of the DIN was the sum of the concentrations of the nitrite (NO_2_-N), nitrate (NO_3_-N), and ammonia (NH_4_-N) salts in the water sample. Following the Specification of Oceanographic Survey (GB 17378.4-2007)^28^, the concentrations of NO_2_-N, NO_3_-N, and NH_4_-N were determined using the Diazo-Azo, zinc cadmium reduction, and sodium hypobromite oxidation methods, respectively.

To analyze the chlorophyll-a concentration, 1 L of seawater (reduced appropriately when the seawater was turbid) was filtered using a glass fiber filter membrane. The film was cryopreserved at a low temperature (< 1 °C). The sample was extracted with acetone at a low temperature under dark conditions, and after 24 h, the chlorophyll-a concentration was measured using a Turner fluorometer.

To determine the petroleum concentration, approximately 500 mL of seawater was collected in a clean brown glass bottle and acidified with concentrated H_2_SO_4_. Within 4 h of sample collection, the seawater sample was extracted using an n-hexane solution. The petroleum concentration in the seawater was determined using ultraviolet spectrophotometry, following the requirements of the Specification of Oceanographic Survey (GB 17378.4-2007)^28^.

### Water discharge from Jiulong River

Monthly water discharge data during 2010–2011 were collected from the website of the Bureau of Hydrology, Ministry of Water Resources, China (http://xxfb.hydroinfo.gov.cn) and previous studies^14,29^. The annual average runoff of Jiulong River was 524.5 m^3^/s in 2010 and 456.7 m^3^/s in 2011. Additionally, the average monthly runoff of Jiulong River was calculated to be 400.4 m^3^/s in August 2010, 286.5 m^3^/s in November 2010, and 658.7 m^3^/s in May 2011.

## Results and discussion

### Hydrography

**Figure 2:**
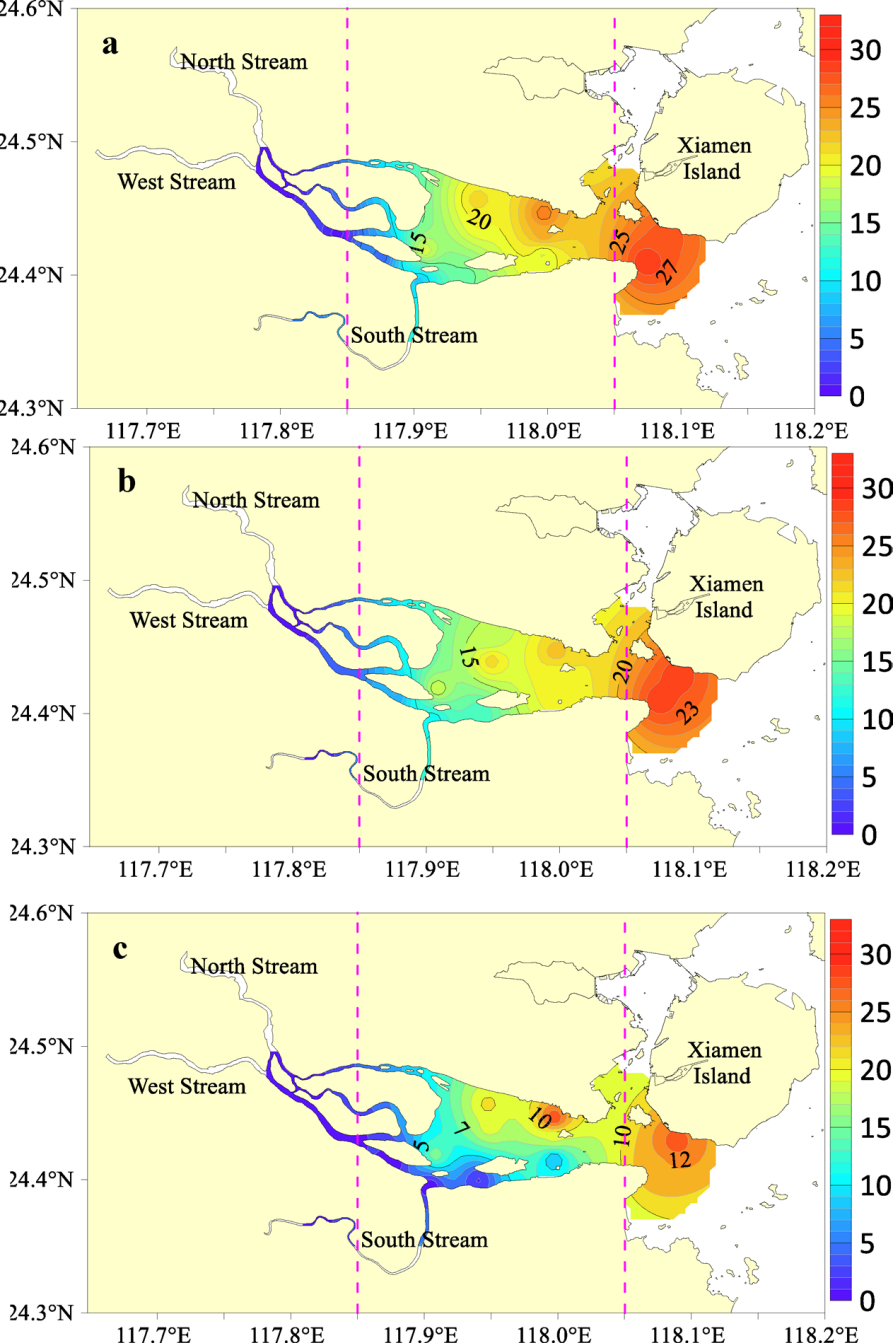

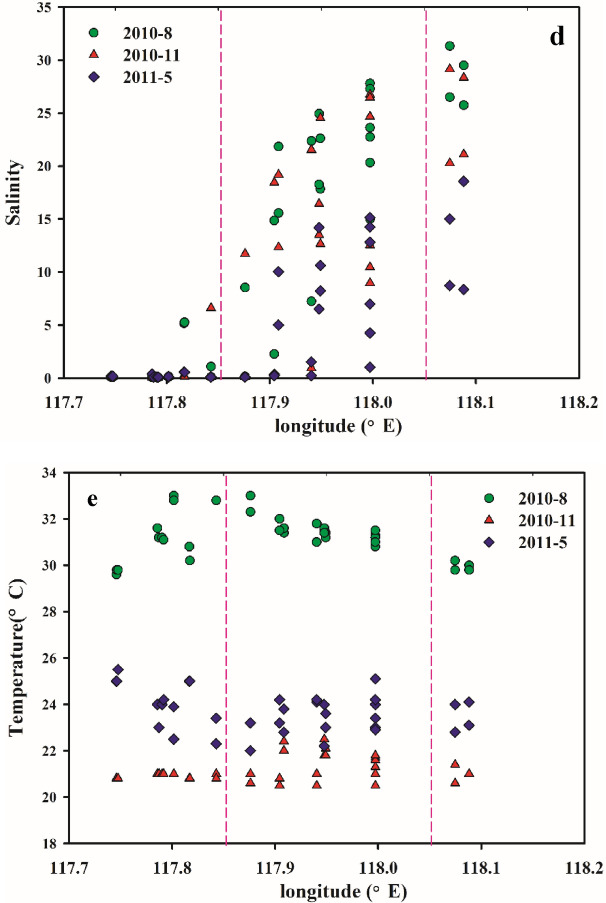
Horizontal distributions of the salinity in (**a**) August 2010, (**b**) November 2010, and (**c**) May 2011 in the JRE. Variations in (**d**) salinity and (**e**) temperature with longitude. These figures were created with Surfer, Version 10 (https://www.goldensoftware.com/products/surfer) (**a–c**) and Sigma-Plot Version 10.0 (Systat Software, Inc., San Jose, California, USA) (**d,e**).

The spatial distribution of the surface salinity along the survey section is shown in Fig. 2. The surface distribution of salinity in the JRE was affected by the diluted Jiulong River water and seawater. In the upper reaches of the estuary, the salinity was extremely low, and the surface salinity of most stations was near 0. In the middle reaches, the mixing of river and seawater was intense, and the salinity range was 0–28. The lower reaches primarily comprised seawater, and the salinity range was 10–32. The surface salinity data of the three cruises demonstrated that the salinity in May 2011 was significantly lower than that in August and November 2010, and it was strongly correlated with the discharge in May 2011. The horizontal distribution was lower in the south and higher in the north, which was primarily related to the JRE tidal characteristics^30^. As illustrated in Fig. 2(e), during the investigation period, the water temperature range was 29.6–33.0 °C in August 2010, with an average of 31.2 °C; 20.5–22.5 °C in November 2010, with an average of 21.2 °C; and 22.0–25.5 °C in May 2011, with an average of 23.7 °C. Overall, the temperature variation trend during each cruise was not significantly different.

### TOC distribution and seasonal variation

#### TOC concentration

The TOC concentration range in the JRE in 2010–2011 was 0.73–8.03 mg/L, with an average of 2.07 mg/L (Table 1). Overall, the average TOC concentration in the JRE was lower than that in the Yangtze River, Pearl River, and Yellow River estuaries. The TOC concentrations were higher than those in the Southern Yellow Sea and Taiwan Strait but were comparable to those in Guangxi Bay, Daya Bay, and Liusha Bay. As displayed in Table 2, the TOC concentration in the surface seawater widely ranged from 0.73 mg/L to 8.03 mg/L, with an average of 2.13 mg/L, while that in the bottom seawater ranged from 0.76 mg/L to 5.32 mg/L, with an average of 1.94 mg/L.

**Table 1:** TOC concentration by oceanic setting.

Sea area	TOC concentration (mg/L)	Sea area	TOC concentration (mg/L)
JRE	0.73–8.03 (2.07)	Daya Bay	1.30–6.30 (2.78)^8^
Yangtze River Estuary	1.90–13.67 (5.69)^31^	Taiwan Strait	0.54–3.68 (1.20)^32^
Yellow River Estuary	1.68–6.38 (4.98)^33^	Guangxi Bay	0.26–6.22 (2.33)^34^
Pearl River Delta	0.71–6.05 (3.46)^35^	Liusha Bay	0.99–5.44 (2.41)^36^
Southern Yellow Sea	0.91–2.53 (1.66)^37^	Arabian Gulf	0.50–3.60 (1.80)^38^

**Table 2:** TOC concentrations in JRE by season (mg/L).

Sampling level	Summer (2010–8)		Autumn (2010–11)		Spring (2011–5)		Average
	Range	Average	Range	Average	Range	Average	
Surface layer	0.73–1.56	1.19	0.90–2.84	1.63	2.24–8.03	3.66	2.13
Bottom layer	0.76–4.17	1.36	1.28–5.32	1.88	1.78–4.08	2.63	1.94
Water column	0.73–4.17	1.23	0.90–5.32	1.71	1.78–8.03	3.28	2.07

#### TOC seasonal variation

The average TOC concentrations in summer, autumn, and spring were 1.23 mg/L, 1.71 mg/L, and 3.28 mg/L, respectively (Table 2), demonstrating an increasing trend. The surface TOC concentrations in summer and autumn were lower than the bottom seawater TOC concentrations. In contrast, the surface TOC concentrations in spring were higher than the bottom seawater TOC concentrations. During the investigated period, the TOC did not display a vertical distribution trend, and the average surface concentrations were slightly higher than the bottom concentrations.

**Figure 3:**
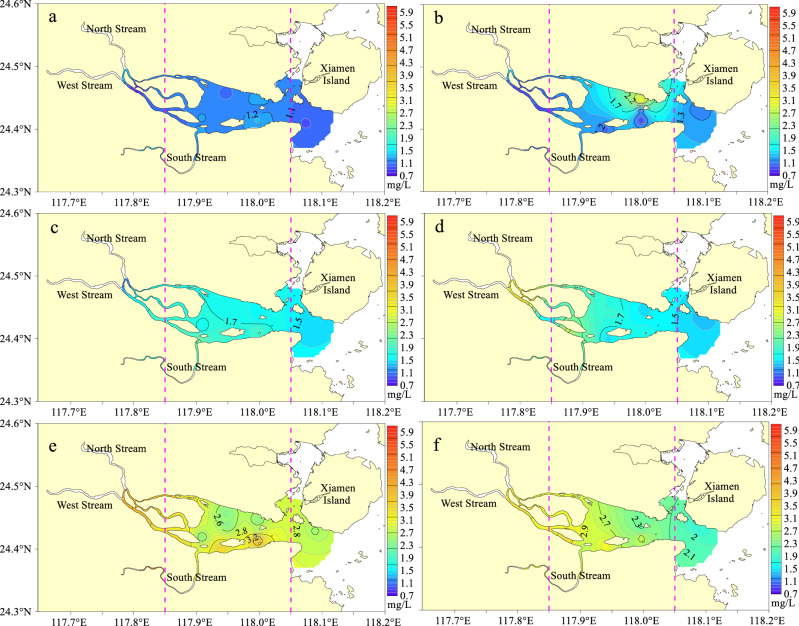
Horizontal distributions of TOC concentrations in the JRE. (**a**) Surface seawater in summer. (**b**) Bottom seawater in summer. (**c**) Surface seawater in autumn. (**d**) Bottom seawater in autumn. (**e**) Surface seawater in spring. (**f**) Bottom seawater in spring. These figures were created with Surfer, Version 10 (https://www.goldensoftware.com/products/surfer).

The TOC concentration distributions are plotted in Fig. 3. In summer (August 2010), the surface TOC contents in the JRE were 0.73–1.56 mg/L, with an average value of 1.19 mg/L, while the bottom seawater TOC concentrations were 0.76–4.17 mg/L, with an average concentration of 1.39 mg/L. The TOC concentration in the bottom layer was higher than that in the surface layer in summer. Additionally, the TOC content in summer was the lowest among those of the three seasons. The maximum summer TOC concentration of 4.17 mg/L was sampled at the A9 station north of the JRE.

In autumn (November 2010), the TOC concentration range in the JRE surface layer was 0.90–2.84 mg/L, with an average concentration of 1.63 mg/L, while that in the bottom layer was 1.28–5.32 mg/L, with an average concentration of 1.88 mg/L. The TOC concentration in the bottom layer was higher than that in the surface layer during autumn. The maximum autumn TOC value was 5.32 mg/L and was sampled at the South Stream monitoring site (A1).

In spring (May 2011), the TOC concentration range in the JRE surface layer was 2.24–8.03 mg/L, with an average concentration of 3.36 mg/L, while that in the bottom layer was 1.78–4.08 mg/L, with an average concentration of 2.63 mg/L. The surface layer TOC concentration was higher than the bottom layer TOC concentration, and the spring samples exhibited the highest average TOC concentration among the samples from the three seasons. The high spring TOC values were 8.03 mg/L, sampled at the South Stream site (N1), and 7.86 mg/L, sampled north of the JRE at the West Stream monitoring site (X1).

### TOC spatial distribution

As can be seen from the TOC spatial distributions of the survey (Fig. 3), the TOC horizontal distribution in the surface and bottom waters of the JRE exhibits strong regularity, and the concentration gradually decreases along the direction of the Jiulong River runoff into the sea. The concentration was higher in the northern and southern parts of the estuary and decreased in the middle section. The horizontal distribution trend was consistent across different monitoring periods. The distributions of the TOC concentration in the upper, middle, and lower reaches of the JRE exhibited different characteristics, and the concentration gradually decreased from the river to the offshore end. The Jiulong River upper reaches are the primary water conveyance regions, where the TOC concentration was the highest, while the Jiulong River middle reaches are the regions where seawater mixing is the most intense, and the TOC concentration varied the most. The nutrient concentration in the lower reaches was the lowest and was controlled by offshore seawater. The vertical distribution of TOC concentration remained consistent, and the average surface concentration was slightly higher than the bottom concentration during the monitoring period.

Sun et al.^32^ found that the TOC content distribution in the Taiwan Strait and its adjacent waters is high near the shore, low far from the shore, high in the north, and low in the middle section, which is consistent with the results of this study. Influenced by river runoff transport, the TOC concentration in the JRE was high. With the weakening of the runoff influence, the TOC amount from the external input into the water body decreases, exhibiting a trend of attenuation from the surface to the bottom and transformation from the external input to biological production^39^.

### Relationship between TOC and estuarine environmental factors

Rivers deposit approximately 4.0 × 10^14^ g of organic carbon into the ocean annually through their estuaries^40^, of which POC and dissolved organic carbon (DOC) account for 40% and 60% of the TOC^41^, respectively. The sources of organic carbon transport in estuaries are diverse and include surface runoff, anthropogenic pollutant discharge, and estuarine phytoplankton photosynthesis^8^. Further, river estuaries exhibit strong physical dynamics and tidal capacities, their salinity gradient varies greatly, the influence of human activity is intense, and their biogeochemical processes are complex^42^; thus, the distribution process of organic carbon in river estuaries varies. Therefore, the TOC content and its spatial and temporal distributions are affected by various factors, including seasonal runoff, topography, hydrodynamics, and biology^43^.

**Figure 4:**
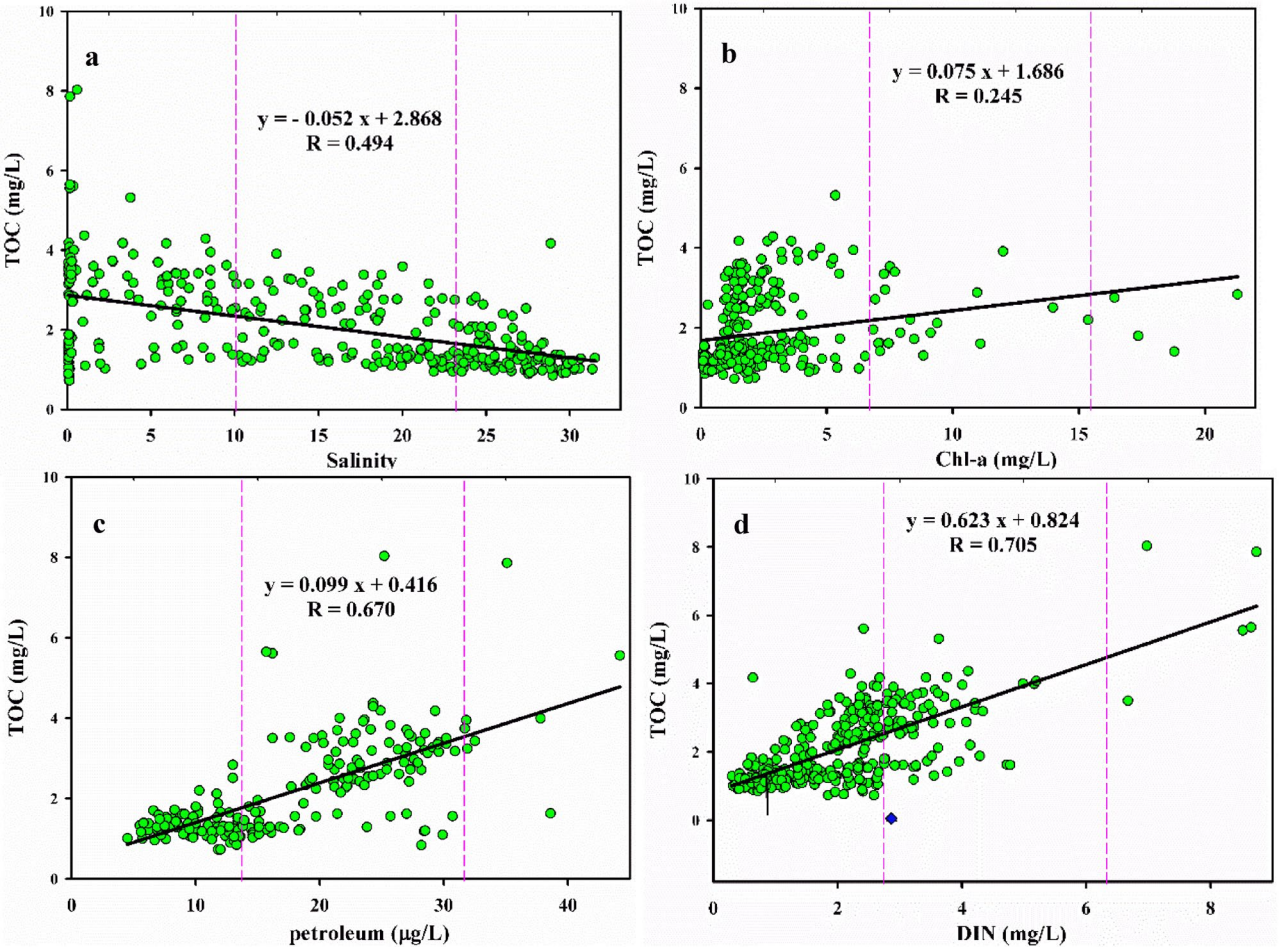
Relationship between TOC concentration and environmental parameters. These figures were prepared using Sigma-Plot Version 10.0 (Systat Software, Inc., San Jose, California, USA).

The TOC concentration in the JRE was significantly negatively correlated with salinity (Fig. 4a). With an increase in the salinity, high-concentration TOC input into the basin gradually decreased, indicating that runoff input from the Jiulong River is the primary factor affecting the TOC concentration distribution and changes in the JRE. Emara^38^ reported that the TOC concentration exhibits a significant negative correlation with salinity due to the influence of exogenous low salinity and high-organic-matter runoff.

The correlation of TOC and chlorophyll-a content in the seawater of the JRE is displayed in Fig. 4b. The figure illustrates a weak positive correlation between TOC and chlorophyll-a content in the JRE, with a correlation coefficient of 0.245. Chlorophyll-a is the primary pigment for phytoplankton photosynthesis and a crucial indicator of marine primary productivity. Marine phytoplankton photosynthesis and biological metabolism are related to TOC production^44^. Studies have demonstrated that phytoplankton exhibit a substantial annual carbon sequestration capacity of over 30 billion^45,46^. Phytoplankton produce high amounts of DOC and POC during photosynthesis and biological metabolic activities, resulting in increased TOC concentrations^41^. POC content is correlated with chlorophyll-a content in the Yangtze estuary^47^, and TOC content was significantly positively correlated with the chlorophyll-a content in Daya Bay from 2006 to 2007^8^. These results indicate that phytoplankton production is a source of TOC. TOC and chlorophyll-a content in the JRE exhibited a weak positive correlation, which is consistent with the results of studies in the Southern Yellow Sea^37^ and Taiwan Strait^32^. This result may be related to the physical processes of seawater and the biological uptake of chlorophyll-a by zooplanktons.

Figure 4c displays the correlation analysis of TOC and petroleum concentrations in the JRE seawater. The TOC concentration in the JRE exhibited a significant positive correlation with petroleum concentration, with a correlation coefficient of 0.675. This result is consistent with those of previous studies^8,38^, which found that TOC concentration increases with an increase in petroleum species because DOC and POC can adsorb and bind organic pollutants through hydrogen bonding, van der Waals forces, hydrophobicity, and other interactions and become their transport carriers^48,49^. The Haicang Port is on the north bank of the JRE, and the Zhaoyin Port is on the south bank. The discharge of oil-bearing wastewater by ships and fishing boats results in a large volume of oil-bearing wastewater near the port areas. Therefore, the input of many petroleum pollutants increases the oil concentration in the JRE seawater, indirectly leading to an increase in the TOC concentration. These findings indicate that the TOC concentration in the JRE may be affected by seasonal river runoff input, phytoplankton, and petroleum pollution.

Nitrogen is a critical nutrient that affects and limits the growth of phytoplankton. It is also the primary cause of eutrophication in estuaries, affecting the change in TOC concentration in water bodies. The relationship between the DIN and chlorophyll-a concentrations in the JRE is positive because high contents of nutrients result in explosive phytoplankton growth, resulting in an increased amount of biomass (chlorophyll-a)^50^. High biomasses (chlorophyll-a) are mineralized into organic matter during downward transport, resulting in high TOC concentrations. Therefore, there was a significant positive correlation (correlation coefficient of 0.705) between TOC and DIN concentrations in the JRE (Fig. 4d), which is consistent with the results of studies conducted in Erhai Lake^51^.

### TOC flux estimation

Estuaries are the confluence of land and sea, and they exhibit biogeochemical processes that significantly affect the flux and process of material transport from rivers to the sea. Therefore, studying TOC transportation and distinguishing its fluxes into estuaries and the sea are necessary for the accurate assessment of river TOC transport and its effects on estuarine and offshore ecosystems^14^.

#### Jiulong River TOC fluxes

The TOC concentration data of the three cruises from 2010 to 2011 and runoff data of the three streams (North Stream, West Stream, and South Stream) were used to calculate the fluxes of the Jiulong River into the sea. Equation (1) was used to calculate the riverine fluxes of TOC into the estuary:

1 $${F}_{i}={C}_{i}\times {Q}_{i}$$

where ${F}_{i}$ is the flux of the TOC, ${C}_{i}$ is the average concentration, and ${Q}_{i}$ is the water discharge of the Jiulong River.

**Figure 5:**
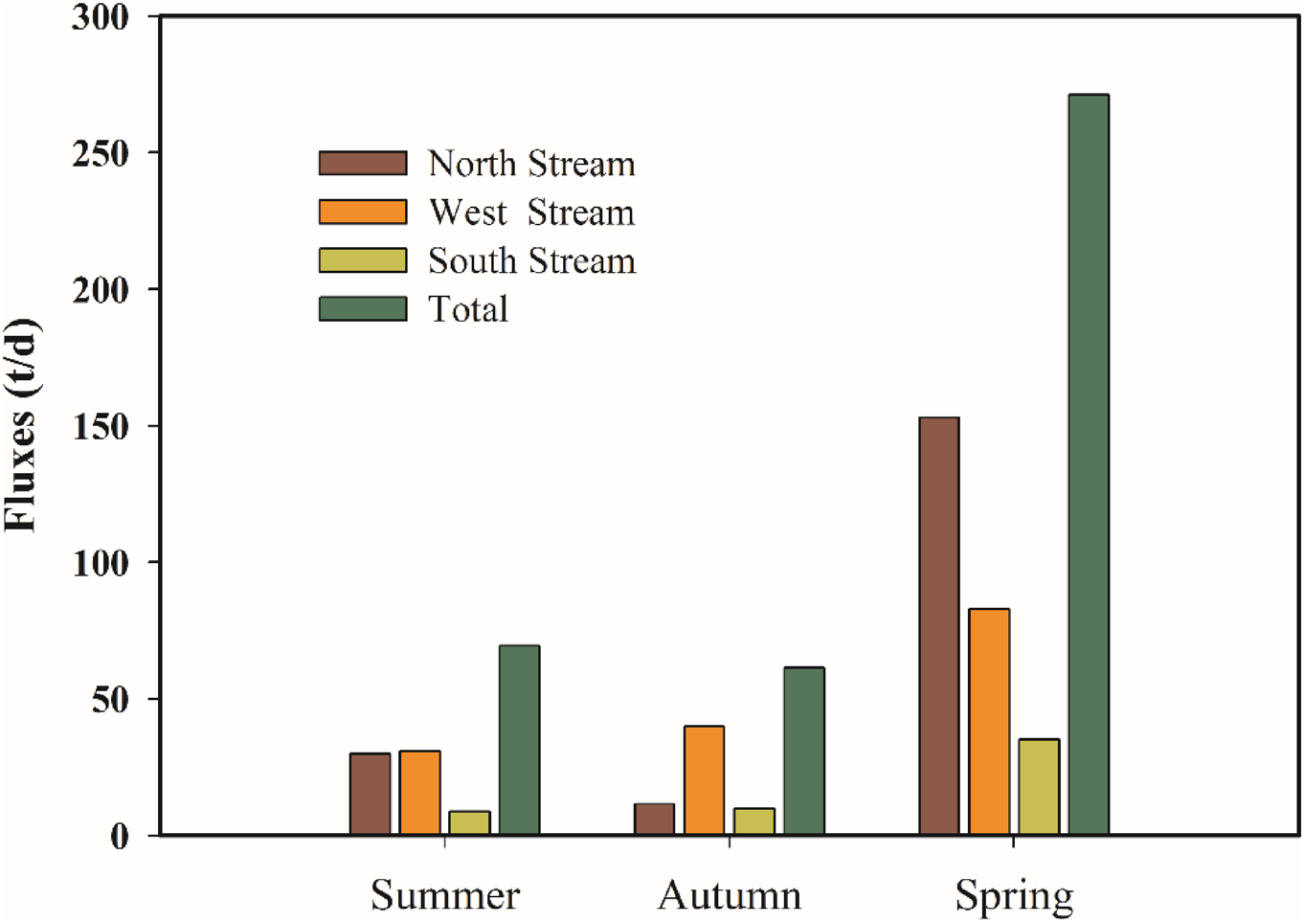
Seasonal variation in TOC fluxes into estuaries for different streams. The figure was prepared using Sigma-Plot Version 10.0 (Systat Software, Inc., San Jose, California, USA).

Figure 5 demonstrates that the JRE flux exhibited significant seasonal differences. In May 2011 (spring), the maximum flux was 271.01 t/d. In August 2010 (summer), the flux into the JRE was 69.40 t/d. In November 2010 (autumn), the flux into the JRE was the lowest at 61.25 t/d. For the three tributaries of the Jiulong River, the fluxes into the North Stream River were in the order of spring > summer > autumn, while the fluxes into the West Stream and South Stream rivers were in the order of spring > autumn > summer. The data comparison in the JRE flux calculation table (Table 3) illustrates that the temporal variation in the TOC flux into the JRE was synchronous with the variation in the runoff. The phenomenon that the TOC flux transported by the river mainly depends on runoff has also been reported in the Yangtze River and the Pearl River estuaries^26^. This finding is consistent with that on nutrient fluxes in the JRE^14^, indicating that runoff is a critical factor affecting the TOC flux in rivers.

**Table 3:** Calculated JRE riverine TOC fluxes by cruise.

Cruise	River	Q (m^3^/s)	C (mg/L)	Riverine fluxes (t/d)	Average C (mg/L)	Total Riverine Fluxes (t/d)
2010–8	North Stream	247.00	1.40	29.88	2.38	69.40
	West Stream	82.88	4.31	30.83		
	South Stream	70.60	1.43	8.69		
2010–11	North Stream	121.00	1.10	11.53	2.53	61.25
	West Stream	94.90	4.87	39.93		
	South Stream	70.60	1.61	9.79		
2011–5	North Stream	437.00	4.05	152.92	5.39	271.05
	West Stream	151.00	6.36	82.93		
	South Stream	70.60	5.77	35.17		

#### Estuarine export fluxes

##### Diurnal variation characteristics of TOC

**Figure 6:**
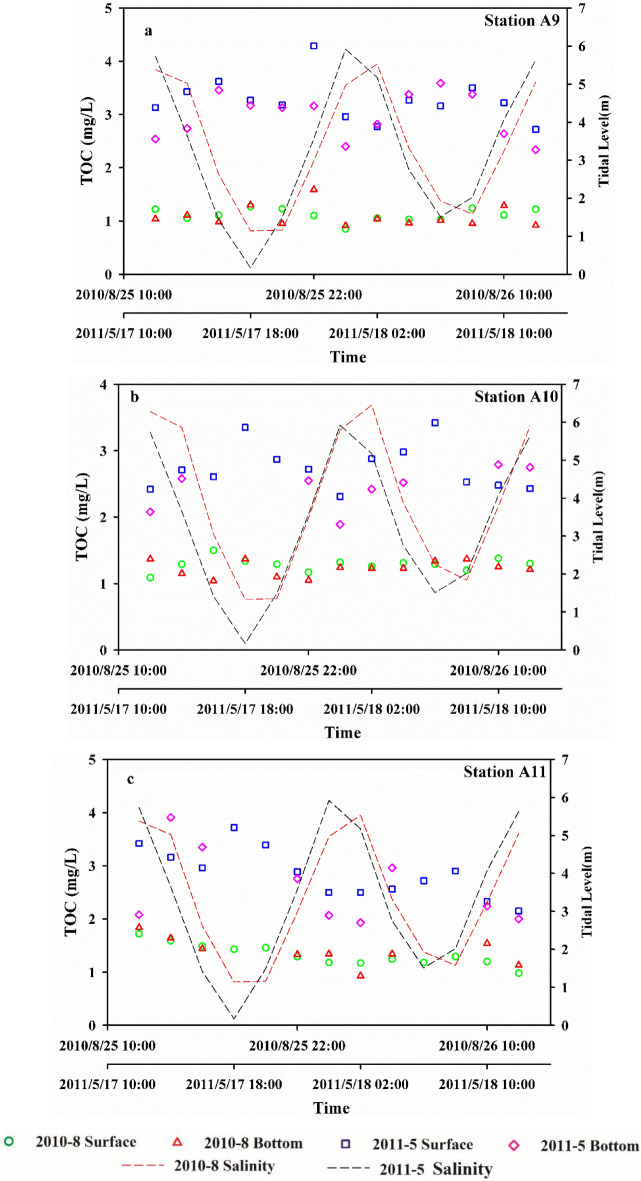
Diurnal variations of TOC concentrations in summer and spring. (**a**) Station A9. (**b**) Station A10. (**c**) Station A11. These figures were prepared using Sigma-Plot Version 10.0 (Systat Software, Inc., San Jose, California, USA).

The TOC concentrations at the surface and bottom of the water column at three stations during one tidal cycle are summarized in Fig. 6. As shown in Fig. 6, the TOC concentrations at the three stations exhibited different interannual, seasonal, and tidal variations. The TOC concentrations in May 2011 were significantly higher than those in August 2010, which is similar to the results of previous large-scale survey data. Higher freshwater inputs in spring 2011 may have led to the greater scour and shorter residence times of TOC in the estuary, reducing the TOC concentration. This postulation is supported by the lower average salinity in 2011 at the JRE.

Continuous tidal cycle observations of salinity demonstrated that, in August 2010, the salinity at stations A9 to A11 was 10.71–30.57, with an average of 24.51. In May 2011, the salinity range was 0.23–27.4, with an average of 14.12. The bottom salinity was higher than the surface salinity in both seasons, and the distribution exhibited certain stratification characteristics. The maximum value of the surface salinity appeared at 12:00 and 2:00 the next day, and the lowest value appeared at 20:00 and 8:00 the next day. The surface salinity appeared as two peaks and troughs within a tidal cycle. The diurnal variation in salinity is consistent with the characteristics of regular semi-diurnal tides in Xiamen Bay and can be used as an indicator of tidal variation.

The TOC concentration exhibited different variation characteristics during the continuous tidal cycle surveys. In August 2010, the TOC concentrations at three stations were 0.85–1.84 mg/L, with an average of 1.23 mg/L. During the tidal cycle, the TOC concentration in the seawater fluctuated, and the concentration was lower in the surface water than in the bottom water. In May 2011, the TOC concentration range of the three stations was 1.89–4.29 mg/L, with an average of 2.86 mg/L. The TOC concentration in the seawater fluctuated during the tidal cycle, and the concentration was lower in the surface water than in the bottom water. The variation in the TOC concentration was negatively correlated with salinity. Overall, the variation characteristics of the TOC concentrations are inconsistent with those of normal semi-diurnal tides in JRE.

##### Diurnal flow variation characteristics

**Figure 7:**
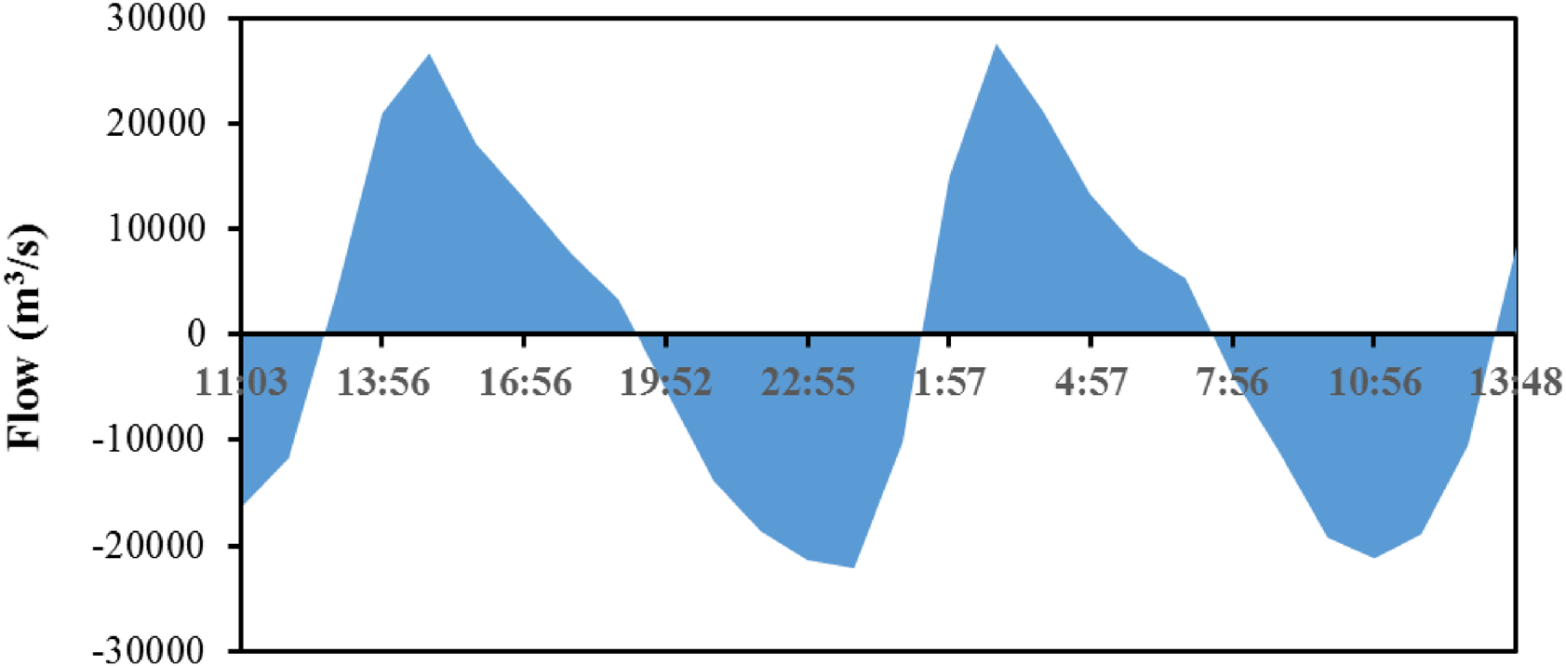
Diurnal flow variations at Jiyu section in the JRE. The figure was prepared using Sigma-Plot Version 10.0 (Systat Software, Inc., San Jose, California, USA).

As illustrated in Fig. 7, the maximum measured ebb tide flow was 27,613 m^3^/s, which appeared at 3:00 on August 26, 2010, and the maximum measured high tide flow was 22,185 m^3^/s, which appeared at 12:00 on August 26, 2010. During the observation period of the flow at the section on the Sunday spring tide, the measured ebb tide volume of the two tide cycles was slightly larger than the measured high tide volume, and the net tidal volume was 27 million m^3^. According to the real-time measurements of tidal current and TOC concentration (Fig. 6), the TOC flux into the sea through the Jiyu section in August 2010 was 47.35 t/d.

##### Export TOC fluxes

**Figure 8:**
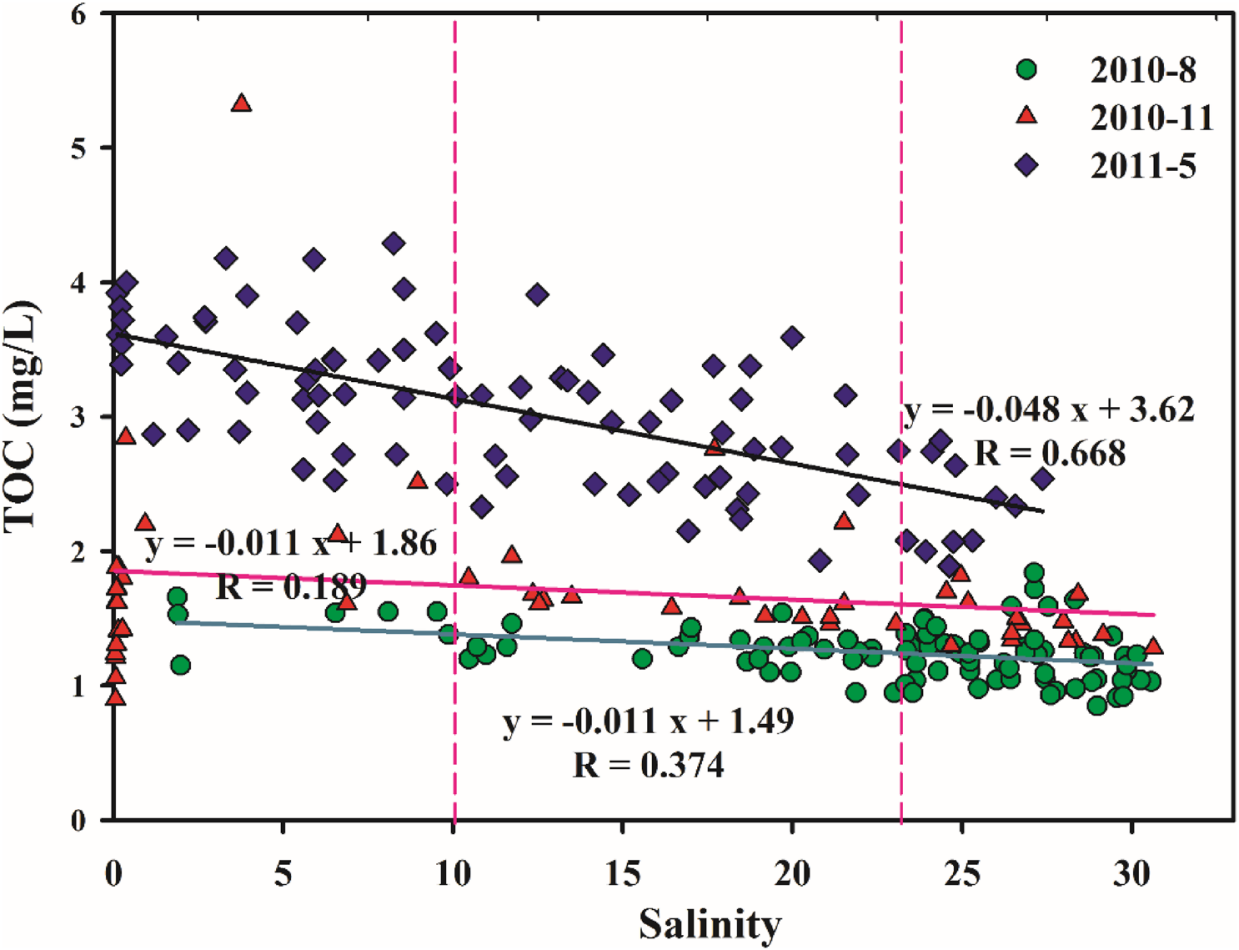
Extrapolation of the effective TOC concentration with salinity in the JRE. The figure was prepared using Sigma-Plot Version 10.0 (Systat Software, Inc., San Jose, California, USA).

The effective concentration method was used to estimate the TOC flux into the sea through the Jiyu section. According to the Officer^53^ method, the TOC concentration of zero salinity (${C}_{0}^{*}$) was defined as the effective concentration of TOC in the sea, which was extrapolated from the linear fitting relationship between the salinity and nutrient concentration in the high-salinity region of the JRE (> 15 PSU). Then the nutrient flux into the sea was obtained by ${C}_{0}^{*}$ multiplied the amount of runoff. Figure 8 displays the calculated relationship of the TOC effective concentration during each cruise investigation. Table 4 illustrates the effective concentration of TOC in the estuarine output and estimated flux into the sea during the survey cruises. Comparing the data on Table 3, it was found that the effective output concentration of TOC in the JRE was less than the mean TOC concentration in the three tributaries measured. The order of the amount of seasonal flux into the sea was spring > summer > autumn, which is consistent with the law of flux into the JRE.

**Table 4:** Estimated effective concentration and export flux of TOC by cruise.

Cruise	Total Q (m^3^/s)	${C}_{0}^{*}$(mg/L)	Seaward export fluxes (t/d)
2010–8	400.40	1.49	51.55
2010–11	286.50	1.85	45.79
2011–5	658.60	3.61	205.42

### Comparison between riverine fluxes and estuarine export fluxes

Table 5 summarizes the estimated TOC fluxes of the JRE during the investigation period. In August 2010, November 2010, and May 2011, the TOC fluxes into the JRE were 69.40 t/d, 61.25 t/d, and 286.50 t/d, respectively, and the fluxes into the sea (calculated as the effective concentration) were 51.55 t/d, 45.79 t/d, and 205.42 t/d, respectively. The fluxes into the sea were less than those into the JRE, indicating that approximately 75% of the TOC from the Jiulong River Basin enters the JRE after undergoing biogeochemical processes. The estuarine flux in August 2010 was 47.35 t/d, which was calculated using the field-measured tidal current flux and continuous TOC concentration and was slightly less than that calculated using the effective concentration method. The Jiulong riverine TOC fluxes into the JRE in 2010 and 2011 were 50.39 × 10^3^ t/a and 46.08 × 10^3^ t/a, and the estuarine export fluxes were 38.46 × 10^3^ t/a and 33.66 × 10^3^ t/a, respectively. These fluxes were calculated based on the weighted runoff.

**Table 5:** Estimated TOC fluxes in JRE.

Project	2010–8 (t/d)	2010–11 (t/d)	2011–5 (t/d)	2010 (× 10^3^ t/a)	2011 (× 10^3^ t/a)
Riverine fluxes	69.40	61.25	286.50	50.39	46.08
Estuarine export fluxes (Tidal flux method)	47.35	–	–	–	–
Estuarine export fluxes (effective concentration method)	51.55	45.79	205.42	38.46	33.66
Percent (%)	74.28	74.76	71.70	76.32	73.05

## Conclusions

The spatial–temporal distributions, fluxes, and seasonal variations in TOC concentration were systematically studied in the subtropical JRE of southeast China, a strong tidal estuary heavily influenced by human activities. The primary conclusions are as follows:

1. In summer 2010, the TOC concentration range was 0.73–4.17 mg/L, with an average of 1.23 mg/L. In autumn 2010, the TOC concentration range was 0.90–5.32 mg/L, with an average of 1.71 mg/L. In spring 2011, the TOC concentration range was 1.78–8.03 mg/L, with an average of 3.28 mg/L. The extent of seasonal variation in the TOC concentration in spring, summer, and autumn occurred in the following order: spring > autumn > summer.
2. The TOC spatial distribution decreased gradually along the Jiulong River runoff into the sea. The TOC concentration was higher in the north and south of the estuary and was lower in the center. Additionally, the TOC concentration was slightly higher in the surface layer than in the bottom layer. The maximum TOC value predominately appeared near the river estuary section.
3. The TOC distribution at the JRE mouth exhibited a significant negative correlation with the salinity, and the petroleum and inorganic nitrogen concentrations presented significant positive and weak correlations with chlorophyll-a content, respectively. These results indicate that terrigenous input affects the TOC distribution in the JRE, and the primary factors affecting the spatial–temporal distribution of the TOC concentration may be the terrain and the influence of biological and port activities.
4. In 2010 and 2011, the riverine TOC input fluxes were 50.39 × 10^3^ t/a and 46.08 × 10^3^ t/a, respectively, and the estuarine export TOC fluxes were 38.46 × 10^3^ t/a and 33.66 × 10^3^ t/a, respectively. The fluxes transported from the estuary to the sea were approximately 75% of those from the Jiulong River, and the estuary significantly affected TOC removal.

Overall, the understanding of the biogeochemical mechanisms of TOC under complex water cycle patterns in subtropical estuarine systems has been significantly improved in this study. However, the highly seasonal and spatial variabilities of the river–estuary–sea systems make TOC budgeting challenging. It is vital to study these variables further regionally to improve our understanding of the global carbon cycle, as regional work is highly sensitive to global scale estimates. Site sampling should be frequently performed seasonally and spatially to study the sources, morphological transformations, and biogeochemical processes of TOC in estuaries.

## Data Availability

The data presented in this study are available on request from the corresponding author.
